# Demographic Disparities in Mortality Trends of Obesity-Related Chronic Kidney Disease Among Adults in the United States From 2018 to 2023

**DOI:** 10.7759/cureus.95300

**Published:** 2025-10-24

**Authors:** Ashir Ahtsham, Uzair Ahmed, Mahrukh Haider, Muhammad Muneeb Alrashid, Nauman Tauqeer

**Affiliations:** 1 Department of Internal Medicine, Lahore General Hospital, Lahore, PAK; 2 Department of General Surgery, Lahore General Hospital, Lahore, PAK; 3 Department of Pathology, Islam Medical and Dental College, Sialkot, PAK; 4 Department of Physiology, Islam Medical and Dental College, Sialkot, PAK

**Keywords:** age-adjusted mortality, chronic disease epidemiology, health disparities, renal outcomes, wonder database

## Abstract

Background: Chronic kidney disease (CKD) is a growing concern in the United States, particularly in the obese population. Despite the rising burden, trends in the mortality rates of obesity-related CKD remain underexplored. The aim of our study was to evaluate demographic disparities in mortality trends among obese individuals with CKD in the United States from 2018 to 2023.

Methods: Death certificate data, obtained from the Centers for Disease Control and Prevention Wide-Ranging Online Data for Epidemiological Research database, involving individuals aged 25 years or older, were studied from 2018 to 2023 for CKD mortality in the obese population. Age-adjusted mortality rates (AAMRs) were analyzed by stratifying the population by sex, age, race, and geographic region.

Results: A total of 9,714 adult deaths, involving individuals aged 25 years or older, from obesity-related CKD were reported between 2018 and 2023. After reaching a peak in 2021 and then falling, the AAMR increased from 0.27 in 2018 to 0.36 in 2023. Mortality rates were slightly higher for men (AAMR: 0.41; 95% confidence interval (CI): 0.39-0.42) than for women (AAMR: 0.38; 95% CI: 0.37-0.39). There were notable racial differences, with Asian adults having the lowest mortality and Native Hawaiians having the highest. Mortality rates increased with age, reaching over 2.0 per 100,000 for those aged 75 years and older. States differed greatly, with New York having the lowest rates and Oklahoma having the highest.

Conclusion: The mortality rates showed a disproportionate rise from 2018 to 2023, with the highest mortality burden in 2021. Trends among men were comparable to those among women. Higher mortality was observed among the elderly, Black populations, and Southern and Midwestern regions. These increasing trends highlight the need for targeted interventions and resource allocation.

## Introduction

The National Kidney Foundation defines chronic kidney disease (CKD) as either a decline in the glomerular filtration rate (GFR) below 60 mL/minute/1.73 m² or the presence of persistent kidney damage for at least three months [1]. Around 35.5 million adults in the United States are estimated to have CKD, while a large number of people remain undiagnosed due to mild initial symptoms [2]. A study shows that multiple factors have led to about 48% of individuals with severe renal dysfunction going undiagnosed [3]. The rising prevalence of CKD has been significantly aided by the progressive rise in obesity all over the world. Obesity has reached pandemic levels over the last few years, and this has been contributing both directly and indirectly to the development and progression of CKD [4]. Obesity may lead to intraglomerular hypertension, fatty deposition in and around the kidneys, podocyte damage, raised adipokine levels, and a considerable disruption of normal neurohormonal regulation, thus leading to a direct effect on the kidneys. In terms of the indirect effect, obesity underlies the majority of cases of type 2 diabetes, which is the leading risk factor in the development of CKD and kidney failure. Hypertension, the second leading risk factor, is also seen to be more common in obese people. According to a recent survey, about 50% of the individuals suffering from CKD in the United States are obese [5].

Despite the growing burden of obesity and CKD, few studies have examined mortality trends across demographic groups, prompting this analysis. Through this study, we aim to analyze the disparities in the mortality trends due to obesity-related CKD in the United States with regard to demographic variations to highlight the importance of targeted interventions to maximize damage control.

## Materials and methods

The data used in the study have been extracted from the Centers for Disease Control and Prevention (CDC) Wide-Ranging Online Data for Epidemiologic Research (WONDER) database, which uses death certificates from the 50 states of the United States to describe the range of mortality causes [6]. The database lets researchers access multiple causes of death (MCD) in addition to the underlying cause of death (UCD), allowing analysis of all relevant conditions that may have contributed to mortality. The International Classification of Diseases (ICD) is used as a basis for identifying and classifying the UCD and the MCD. In our study, MCD Public Use Record death certificates were analyzed using the ICD-10 codes for CKD that correspond to ICD-10 codes under N18 (N18.0, N18.1, N18.2, N18.3, N18.4, N18.5, N18.8, and N18.9) [7]. With the aim of restricting our study to obese people, all the decedents with obesity (>E66, E66.0, E66.1, E66.2, E66.8, and E66.9) were included as a contributing cause to CKD. Beyond the specified ICD-10 codes for CKD and obesity, individuals aged less than 25 years and those of Hispanic origin were excluded from the study due to very limited data. Our study did not require institutional review board approval because it involved the exclusive analysis of government-issued data.

All the available data on CKD-related mortalities in obese people from 2018 to 2023 were extracted and categorized based on different age groups, sex, race, and state. Individuals aged 25 years or more were studied and divided into 10-year age groups to analyze age-related trends. The mortality trends in Whites were extracted and analyzed against those in other races, such as African Americans, Asians, American Indians, and Native Hawaiians, to determine the racial disparities. Similarly, men were compared to women. Additionally, we extracted the state-stratified data available in the dataset.

The relevant parameter used to analyze mortality rates was the age-adjusted mortality rate (AAMR) per 100,000 population; however, crude mortality rates (CMRs) were used to depict the mortality burden across age groups because AAMRs were unavailable for those age groups in the database. AAMRs were determined by standardizing the obesity-related CKD deaths to the 2000 U.S. population [6]. These AAMRs were then analyzed using the Joinpoint Regression Program (version 5.4.0.0, April 2025; IMS, Inc., Calverton, Maryland), a statistical analysis tool designed to identify points where a significant change in trend occurs within the provided dataset [8]. The software was used to analyze trends in AAMRs across various domains and to outline any significant variations in those trends. Model selection was performed using the Monte Carlo Permutation method, the default criterion in the software. Corresponding p values were also derived from the same permutation method. A p value less than 0.05 was considered significant. Where applicable, 95% confidence intervals (CIs) were used as the determinant of statistical significance.

The findings of the Joinpoint Regression were then displayed as annual percentage changes (APCs) with 95% CIs and corresponding p values for the mortality rates in the selected cohort. State-level AAMRs were visualized using choropleth maps created in R (RStudio 2025.05.1+513 "Mariposa Orchid" Release; Posit Software, Boston, MA) with the tmap and sf packages [9]. Portions of the description of results were generated with the assistance of ChatGPT (OpenAI, San Francisco, California) and subsequently reviewed and edited by the author.

## Results

Between 2018 and 2023, a total of 9,714 obesity-related CKD deaths were recorded among adults aged 25 years or older. Of these, 64.6% occurred in medical facilities, 10.4% in nursing homes or long-term care facilities, 19.5% at home, and 3.7% in hospices. Exact information was not available for 1.6% of the deaths. The AAMRs for obesity-related CKD in adults increased from 0.27 in 2018 to 0.36 in 2023. During 2018-2021, the AAMR showed an upward trend, whereas from 2021 to 2023, a decline was observed (Table 1).

**Table 1:** Deaths, overall AAMRs, and APC of obesity-related chronic kidney disease mortality in adults in the United States from 2018 to 2023 AAMR: age-adjusted mortality rate; APC: annual percentage change; CI: confidence interval ^*^Crude mortality rates

Variable	Deaths (n)	AAMR (95% CI)	APC (95% CI)	p value
Overall	9,714	0.4 (0.39 to 0.41)	6.43 (-15.95 to 34.75)	0.50
Sexual disparities				
Female	5,296	0.38 (0.37 to 0.39)	5.12 (-11.13 to 24.34)	0.46
Male	4,418	0.41 (0.39 to 0.42)	6.04 (-10.91 to 26.21)	0.40
Racial disparities				
American Indian or Alaska Native	109	0.46 (0.37 to 0.55)	62.5	-
Asian	66	0.06 (0.05 to 0.08)	-	-
Black or African American	1,764	0.66 (0.62 to 0.69)	9.46 (-12.52 to 36.95)	0.33
Native Hawaiian or Other Pacific Islander	63	1.27 (0.97 to 1.63)	-	-
White	7,646	0.38 (0.37 to 0.39)	6.06 (-14.08 to 30.91)	0.48
Age disparities (years)				
25-34	145	0.1 (0.0 to 0.1)^*^	-3.18 (-28.28 to 30.69)	0.69
35-44	411	0.2 (0.1 to 0.2)^*^	5.59 (-17.00 to 34.32)	0.56
45-54	1,041	0.4 (0.4 to 0.5)^*^	6.76 (-15.08 to 34.22)	0.47
55-64	2,087	0.8 (0.8 to 0.9)^*^	4.47 (-13.23 to 25.78)	0.55
65-74	2,982	1.5 (1.5 to 1.6)^*^	4.59 (-17.04 to 31.85)	0.62
75-84	2,217	2.2 (2.1 to 2.3)^*^	7.13 (-11.24 to 29.31)	0.37
85+	813	2.1 (2.0 to 2.3)^*^	14.13 (-2.36 to 33.41)	0.08

**Figure 1:**
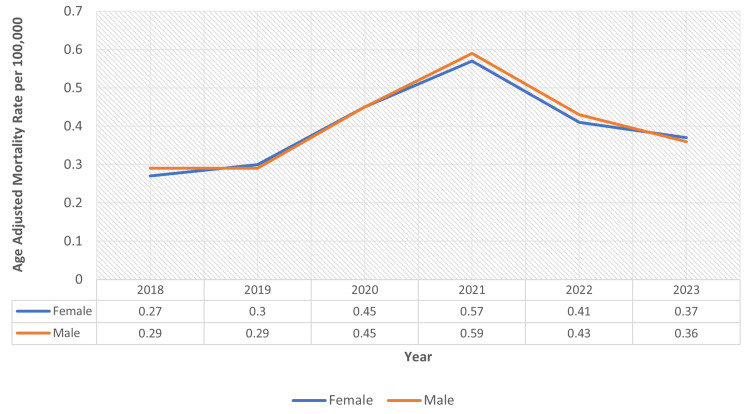
Sex-stratified age-adjusted mortality rates of obesity-related chronic kidney disease

Throughout the study duration, men demonstrated slightly higher AAMRs than women (overall AAMR men: 0.41; 95% CI: 0.39-0.42; overall AAMR women: 0.38; 95% CI: 0.37-0.39). The AAMR for men showed an increase from 0.27 in 2018 to 0.37 in 2023, whereas for women, the AAMR increased from 0.29 in 2018 to 0.36 in 2023 (Figure 1).

Among men, the AAMR showed an upward trajectory during 2018-2021, followed by a decline from 2021 to 2023. Across the entire study period (2018-2023), the APC was 6.04 (95% CI: -10.91 to 26.21; p = 0.40). For women, the AAMR also increased during 2018-2021 and subsequently declined between 2021 and 2023, with an overall APC for 2018-2023 of 5.12 (95% CI: -11.13 to 24.34; p = 0.46).

**Figure 2:**
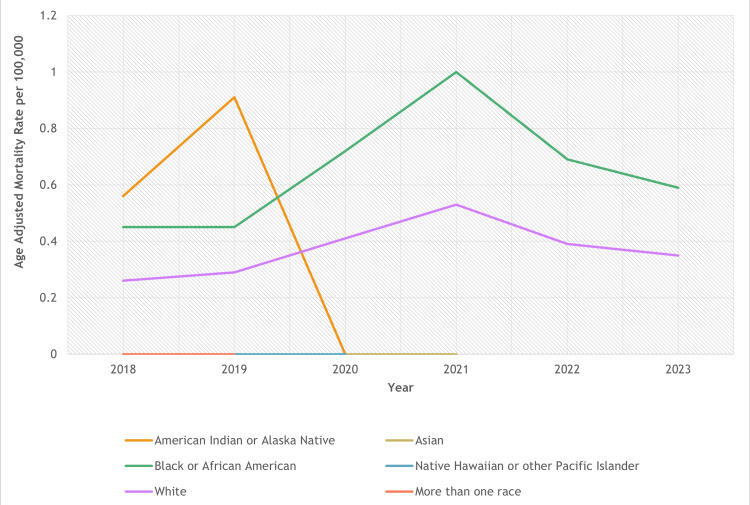
Race-stratified age-adjusted mortality rates of obesity-related chronic kidney disease

Between 2018 and 2023, there were noticeable disparities in CKD mortality related to obesity across different racial and ethnic groups. The highest overall AAMR was found among Native Hawaiian or Other Pacific Islander adults (1.27; 95% CI: 0.97-1.63), followed by Black or African American adults (0.66; 95% CI: 0.62-0.69) and American Indian or Alaska Native adults (0.46; 95% CI: 0.37-0.55). White adults had an AAMR of 0.38 (95% CI: 0.37-0.39), whereas Asian adults recorded the lowest rates at 0.06 (95% CI: 0.05-0.08) (Figure 2).

Regarding trends over time, Non-Hispanic Black or African American adults exhibited a slight, nonsignificant (p = 0.33) rise throughout the study period (APC: 9.46; 95% CI: -12.52 to 36.95). Likewise, White adults also experienced a minor nonsignificant (p = 0.48) increase in mortality (APC: 6.06, 95% CI: -14.08 to 30.91). The estimated APC for American Indian or Alaska Native adults was 62.5, although precision was constrained due to a limited sample size. Among Asian and Native Hawaiian or Other Pacific Islander adults, unstable patterns were noted, and no calculable APCs were available due to low case counts.

**Figure 3:**
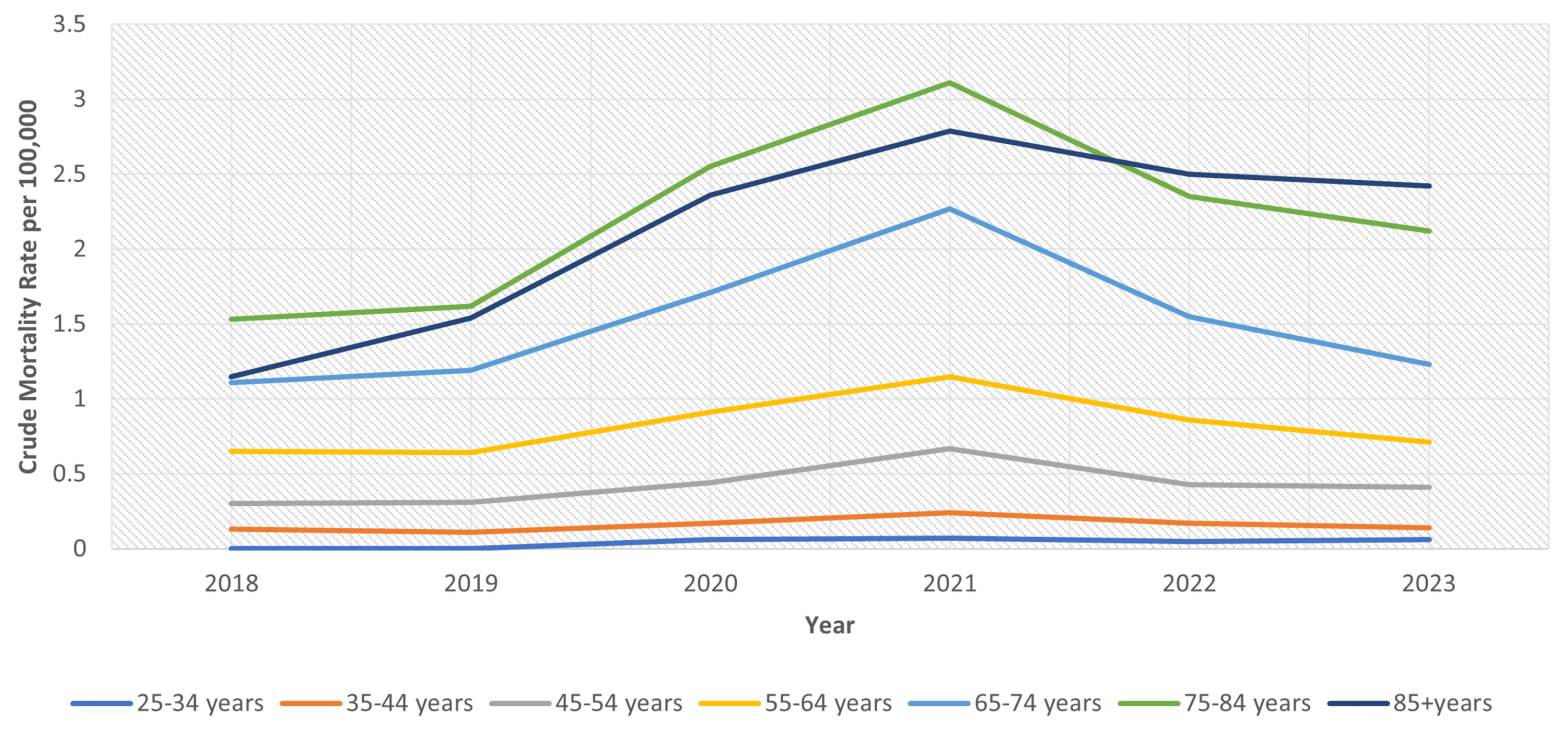
Age-stratified crude mortality rates of obesity-related chronic kidney disease

Across all age categories, CMRs associated with obesity-related CKD rose with increasing age. The lowest burden was noted in individuals aged 25-34 years (CMR: 0.1 per 100,000; 95% CI: 0.0-0.1), whereas the highest rates were seen in those aged 75-84 years (CMR: 2.2; 95% CI: 2.1-2.3), closely followed by individuals aged 85 years and older (CMR: 2.1; 95% CI: 2.0-2.3). Joinpoint regression analysis showed varied trends among different age groups. Younger adults (25-34 years) demonstrated a nonsignificant reduction (APC: -3.18; 95% CI: -28.28 to 30.69), whereas the 35-44 years cohort exhibited a slight, albeit nonsignificant, increase (APC: 5.59; 95% CI: -16.99 to 34.32). In contrast, adults aged 45 years and older generally displayed positive APCs, with the most pronounced, yet nonsignificant, rise occurring among individuals aged 85 years and older (APC: 14.13; 95% CI: -2.36 to 33.41) (Figure 3).

**Figure 4:**
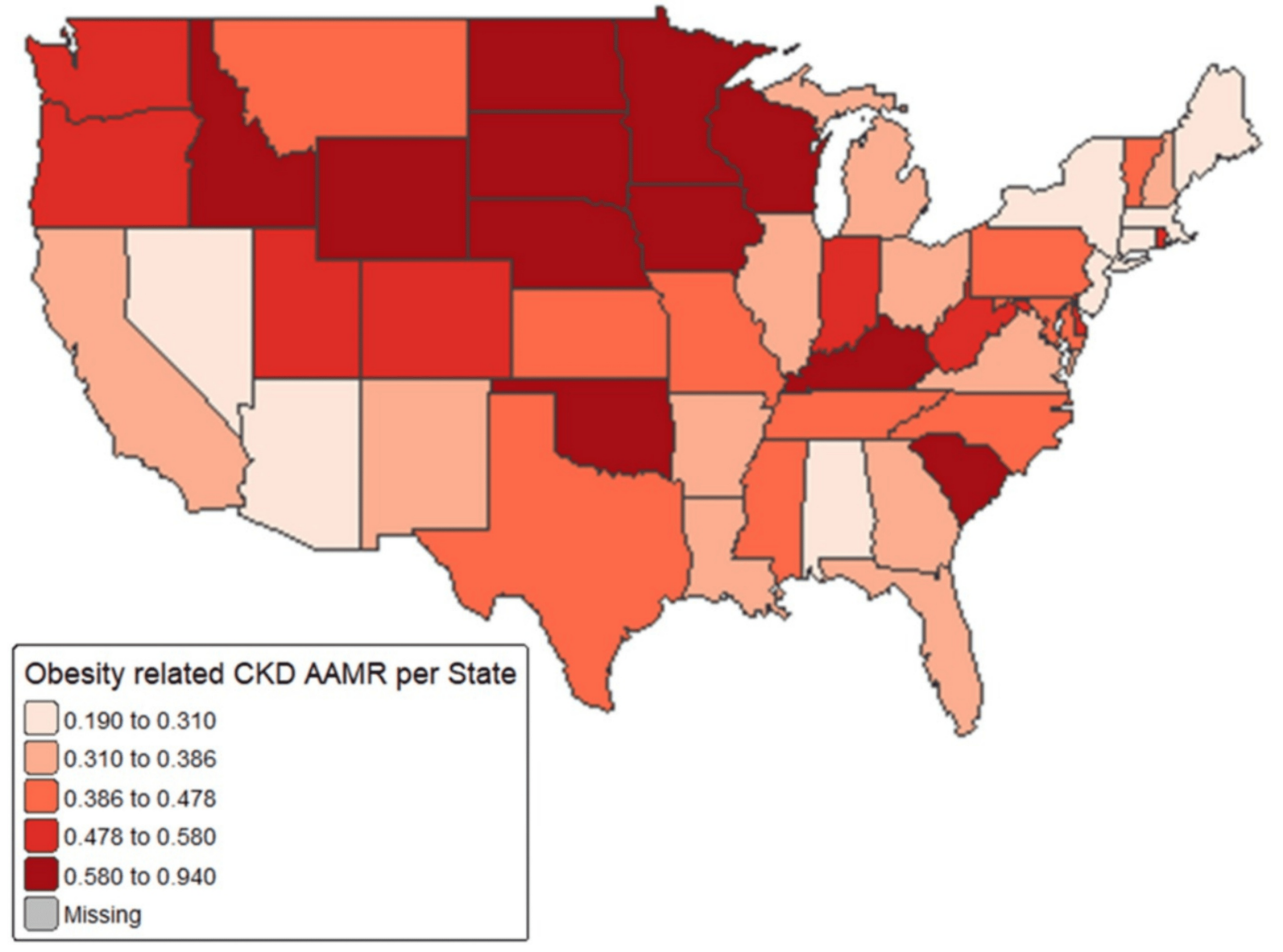
State-stratified age-adjusted mortality rates of obesity-related chronic kidney disease CKD: chronic kidney disease; AAMR: age-adjusted mortality rate Image credit: This is an original image created by the author Ashir Ahtsham

These results underscore the unequal mortality burden faced by older age groups, especially those aged 65 years and older, where CMRs surpassed 1.5 per 100,000 and APCs indicated upward trends. Significant geographic disparities in obesity-related CKD mortality rates were identified across U.S. states from 2018 to 2023. The states with the highest AAMRs included Oklahoma (0.94), Wisconsin (0.74), South Carolina (0.72), South Dakota (0.68), Nebraska (0.67), Minnesota (0.64), and North Dakota (0.63). Additionally, states such as Indiana (0.55), Kentucky (0.59), and West Virginia (0.56) also exhibited rates exceeding the national average. In contrast, the lowest mortality rates were found in New York (0.19), Massachusetts (0.22), Hawaii (0.23), New Jersey (0.24), and Connecticut (0.22), all with AAMRs below 0.25. States with intermediate rates included large states such as California (0.31), Texas (0.44), and Florida (0.31). Data for Alaska were deemed statistically unreliable due to insufficient sample sizes (Figure 4).

## Discussion

Using the six-year data from the CDC WONDER, several key findings have been reported in this study in terms of mortality trends due to obesity-related CKD. Although the APCs were not statistically significant across the subgroups (Table 1), the overall direction of change suggests that mortality from obesity-related CKD has not improved in recent years. An overall rise in mortality was observed in all age groups above 34 years across the six-year period, with people aged over 75 years showing a relatively greater increase. The highest rates were reported in the year 2021. These rising trends were consistent among both men and women. Non-Hispanic Blacks showed the highest AAMRs among all the studied races. When analyzing the different states, the highest AAMRs were found to be reported in Oklahoma, whereas New York showed the lowest rates.

Obesity-related CKD mortality demonstrated a rise in AAMRs from 2018 to 2021, with a recent decline seen from 2021 to 2023. This rising trend in the initial years coincides with the COVID-19 pandemic, which largely contributed to increased mortality in people with pre-existing CKD. As suggested by several studies, people with CKD were at very high risk for acquiring COVID-19 infection, and the pre-existing renal condition significantly worsened their prognosis when infected [10]. This temporal correspondence suggests that pandemic-related factors may have contributed to the short-term rise in mortality from 2018 to 2021, whereas the observed decline in mortality after 2021 may, in part, reflect the nationwide COVID-19 vaccination rollout and improved access to preventive healthcare, which likely reduced the mortality risk among individuals with preexisting chronic conditions, including CKD. However, as the data are ecological and limited to death certificate records, causal inference cannot be made.

According to our analysis, the gradual rise in the AAMRs across the six-year period in men was almost similar to that in women, with both genders peaking in 2021. While both sexes demonstrated an upward trend between 2018 and 2021, followed by a decline thereafter, these changes were not statistically significant (women: p = 0.46; men: p = 0.40). Previous studies indicate a more rapid decline in GFR leading to a higher progression rate of CKD in men compared with women, which is mainly attributed to the renoprotective role of estrogen. Despite a faster progression to end-stage renal disease in men, mortality rates may not always be higher due to several interconnected factors [11,12]. Women, being less likely to be referred for a kidney transplant, may serve as a contributing factor for mortality due to end-stage renal disease, despite the slower progression of the disease [13].

A divergent pattern was observed when studying the age-related trends. With the exception of young adults (25-34 years), mortality rates increased across all age groups; however, the APCs in all age groups remained statistically nonsignificant (Table 1). The steepest rise was seen in older adults, particularly those aged 85 years and older. These rising trends in mortality with progressing age may be attributed to a higher prevalence of modifiable risk factors, such as diabetes and hypertension, in the older group of individuals [14]. Prolonged obesity exposure in elderly adults may worsen metabolic, hemodynamic, and inflammatory disruption, reducing prognosis [15]. Postmenopausal hormonal changes in women may also account for a relatively higher obesity-related CKD mortality with increasing age, owing to the beneficial effects of estrogen in kidney protection [16]. Evidence suggests that younger adults with obesity-related kidney disease tend to show better outcomes when provided with treatment modalities compared with older age groups with similar conditions [17]. A retrospective study carried out in the United States reported that 96.2% of individuals aged below 45 years with CKD were either receiving or preparing for renal replacement therapy, whereas the percentage dropped to 53.3% in people aged 85 years and older [18].

The results also demonstrated a rise in the race-stratified mortality rates, with African Americans showing a more substantial rise in AAMRs compared with Whites. Although the APCs for racial groups were not statistically significant (Table 1), these findings may be attributed to a faster progression of the disease in these African Americans due to biological, social, and genetic factors, including APOL1 risk alleles [19]. Structural and socioeconomic parameters may also serve as a contributing factor for a comparatively higher burden of CKD in Black people, with existing disparities in the United States and other regions of the world reflected in broad studies in terms of CKD [20]. Apart from a greater burden of kidney disease, Non-Hispanic Blacks are more likely to be affected by obesity compared with Non-Hispanic Whites in the United States, thus resulting in higher AAMRs for obesity-related CKD [6].

In addition, significant geographical variations in obesity-related CKD mortality were observed. States in the South and Midwest were found to be associated with the highest AAMRs across the six-year period, whereas the Northeastern states exhibit very low AAMRs. These disparities are consistent with adult obesity prevalence across various states of the United States [6].

Taken together, the analysis suggests a rise in obesity-related CKD mortality in the studied demographic groups, with disproportionate increases among the elderly and racial minorities. This highlights the need for targeted public health interventions, including increased awareness regarding obesity and timely screening of chronic kidney conditions in high-risk populations, such as the elderly, racial minorities, and Southern and Midwestern populations. Furthermore, equitable access to treatment modalities should be encouraged.

Strengths and limitations

Strengths of this study include the use of a comprehensive national mortality database, standardized AAMR calculations, and stratified trend analysis across multiple demographic groups. However, several limitations of our study should be considered. First, only a short observation window (2018-2023) was used, which may limit the stability of trends in some subgroups. Second, we relied on death certificate coding, which may introduce misclassification of CKD and obesity as the cause of death. Moreover, obesity classification on death certificates is based on diagnostic coding and may not capture differences in metabolic health. Third, the available data may lack information regarding the screening techniques, medical treatments, and interventions. Fourth, interaction effects between demographic factors, such as sex and race, were not assessed due to small subgroup sample sizes, which may limit exploration of intersectional disparities. Fifth, because CMRs are unadjusted, age-group comparisons should be interpreted cautiously. Additionally, no data on relevant clinical variables were available to help us better understand the phenotypic variations of the underlying and contributing cause.

## Conclusions

Our study indicated a rise in mortality from obesity-related CKD among American adults aged 25 years or older, from 2018 to 2021, followed by a decline in the next two years. This burden appeared more pronounced among the elderly, racial and ethnic minorities, particularly Black and Native Hawaiian populations, and Southern and Midwestern regions, though differences were not statistically significant. Mortality rates among men and women were comparable. These results highlight the increasing influence of obesity on CKD outcomes and necessitate the urgent need for targeted interventions, including early detection of CKD through screening, awareness regarding obesity and CKD, and equitable access to effective treatments, including dialysis and transplantation. To effectively tackle obesity-related CKD as a complex public health challenge, it is essential to implement coordinated strategies that involve both healthcare and policy sectors. Targeted interventions focused on elderly adults, racial and ethnic minorities, and residents of Southern and Midwestern states, combined with research that considers both clinical and social health determinants, can be vital in reducing mortality rates and addressing disparities in obesity-related kidney health.
